# Genetic Engineering in Animal Models

**Published:** 1995

**Authors:** Susanne Hiller-Sturmhöfel, Barbara J. Bowers, Jeanne M. Wehner

**Affiliations:** Susanne Hiller-Sturmhöfel, Ph.D., is a science editor of Alcohol Health & Research World. Barbara J. Bowers, Ph.D., is a research associate in the Department of Psychiatry, Indiana University, Indianapolis, Indiana. Jeanne M. Wehner, Ph.D., is a professor at the Institute for Behavioral Genetics at the University of Colorado at Boulder, Boulder, Colorado

**Keywords:** animal model, animal strains, AOD dependence, research, genetics and heredity, gene, RNA

## Abstract

Multiple genetic and environmental factors influence the development of alcoholism. To evaluate the contributions of individual genes to the development of alcoholism in living organisms, rather than in tissue-culture experiments, researchers have begun to use new genetic technologies in laboratory animals. These techniques include generating transgenic mice, in which a foreign gene is inserted permanently into the animal’s genetic material; generating knockout mice, in which a gene is permanently inactivated; and using antisense ribonucleic acid (RNA) treatment, which allows the temporary inactivation of individual genes. Although not yet widely used in alcohol research, these technologies may allow researchers to study important questions and gain new insights into the causes and consequences of alcoholism.

The use of animal models to elucidate causes and mechanisms of human diseases and to develop new treatment approaches is a mainstay of modern biological research, including alcohol research. Laboratory animals, such as rats and monkeys, are used to model drinking behavior, to study alcohol-induced damage to different organs, and to analyze the brain chemistry involved in mediating alcohol’s effects.

An important focus of current alcohol research is to identify genes that contribute to alcohol drinking behavior and its consequences. The functions of these genes, that is, the mechanisms through which the genes exert their effects, are difficult to study. Genetic studies with isolated cells and tissues, which have been successful in analyzing the causes and consequences of other diseases, cannot reflect behavioral responses, such as those that occur in alcoholism. Conversely, genetic studies in whole organisms, whether laboratory animals or humans, are difficult to conduct and interpret because scientists and researchers believe that alcoholism is influenced by both genetic and environmental factors. In addition, alcoholism is a polygenic disease (i.e., many genes play a role in its development), making it hard to determine the contribution of each individual gene.

Recent progress in employing genetic engineering technologies to develop new animal models eventually may enable researchers to overcome some of these difficulties and to study the role of individual genes and their products in the development of alcoholism. These technologies allow the insertion of foreign genes, the permanent inactivation of specific genes, and the temporary elimination of particular gene products in a living organism. Using these approaches, researchers can evaluate the impact of individual genes on the development of a disorder such as alcoholism.

This article describes three of these new technologies: transgenic mice, knockout mice, and treatment with antisense ribonucleic acid (RNA). Although these approaches have not been used widely in alcohol research, their use in other research areas indicates both their potential applications and limitations in the alcohol field.

## Transgenic Mice

In transgenic animals, a foreign gene is integrated permanently into the animal’s genetic material, the DNA,^1^ in both the reproductive (i.e., germ) cells and the nonreproductive (i.e., somatic) cells, leading to the expression and propagation of the gene across future generations. This technique has been used primarily to evaluate the role of specific genes during fetal development or to mimic human diseases in animals. In the latter case, scientists introduce into an animal a human gene that is known to cause a disorder and then study the animal’s development of the disease. Examples of human diseases that have been studied in transgenic animals include cystic fibrosis (Snouwaert et al. 1992) and muscular dystrophy (Vincent et al. 1993). Studying the mechanism of the development of a disease in more detail may enable scientists to devise better prevention or treatment approaches and subsequently evaluate them in these animals.

Similarly, genes that are known or suspected to contribute to alcoholism could be introduced into transgenic animals. It may be more difficult, however, to measure a gene’s effect on a behavior, such as alcohol consumption, than on a specific bodily function or biochemical process. Consequently, alcohol researchers are still evaluating the potential of transgenic animals for their studies.

### Creating a Transgenic Mouse

Although researchers can use several mammalian species to create transgenic animals, they primarily use mice. Mice can be bred easily, they have a short generation period, they bear many pups per litter, their embryos can be manipulated easily during experiments, and their genes have been studied extensively (Lovell-Badge 1985).

Before being introduced into a mouse or any other animal, the foreign gene must first be identified and isolated from its original organism (e.g., from the DNA of human cells). Next, many identical copies of the gene are chemically synthesized, which then are injected into mouse embryos.

**Figure 1:**
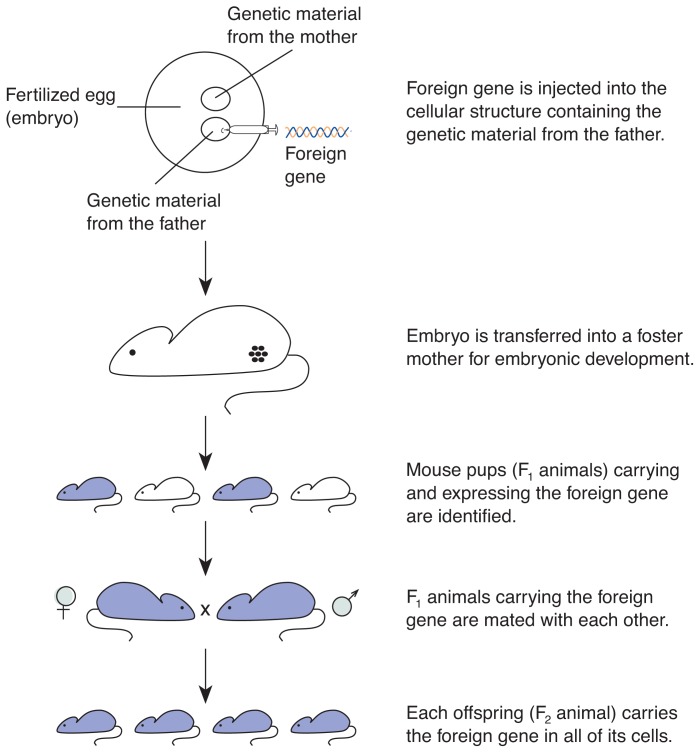
General procedure for the generation of transgenic mice.

To create mouse embryos, eggs of laboratory mice are fertilized in a test tube with mouse sperm. The fertilized egg, or embryo, contains two sets of DNA, each of which is contained in a separate structure called a pronucleus. One DNA set comes from the mother (i.e., the female pronucleus); the other set comes from the father (i.e., the male pronucleus). At this stage, the foreign gene is added by injecting the DNA directly into the male pronucleus with a very fine glass needle (figure 1) (also see Gordon et al. 1980). Although this procedure may seem straightforward, its challenges become obvious when one considers that the whole embryo is only about 0.1 mm in diameter.

In the 50 to 90 percent of the injected embryos that survive this procedure (the success rate depends on the scientist’s skill), the foreign gene integrates into the embryo’s DNA, and the embryo continues to develop normally. The two pronuclei fuse, and the cell begins to divide. The embryos are implanted into surrogate mothers, where about 10 to 30 percent of the embryos develop to term. The mouse pups then can be tested to determine whether they have integrated the foreign gene into their DNA and whether they actually express the gene (i.e., synthesize the protein that is the gene product). (For more information on some of the basic processes of gene expression, see sidebar, p. 208.)

**From DNA to Protein: How Genetic Information Is Realized**

All the genetic information that is necessary to create and maintain an organism is encoded in long, thread-like DNA molecules in the nucleus of each of the organism’s cells. But how is this information converted into the proteins that compose a significant portion of the cell’s components and which drive most chemical reactions in the body? This conversion, which also is referred to as gene expression, is a complex biochemical process that consists of several steps occurring in the cell nucleus and in the cytoplasm. To better understand how gene expression works, it helps to review briefly the chemical structure of DNA. The characteristic design of DNA molecules is the basis for the reactions involved in gene expression.

The building blocks of DNA, the nucleotides, are sugar molecules linked to organic bases. DNA includes four different organic bases: adenine (represented by the letter A), cytosine (represented by the letter C), guanine (represented by the letter G), and thymine (represented by the letter T). The order in which they are arranged specifies which amino acids will be linked to form a protein. Because more than four amino acids exist and are necessary to produce a protein, a triplet of three nucleotides represents (i.e., codes for) one specific amino acid in the final protein. For example, the nucleotide triplet ATG codes for the amino acid methionine, and the triplet TGG codes for the amino acid tryptophan. The section of a DNA molecule containing the information needed to make one specific protein is called a gene.

DNA is a double-stranded molecule: Two chains of nucleotides face each other and are connected through specific bonds (see figure 3 of the main article). Because of the nature of these bonds, each nucleotide can bind to only one other particular nucleotide. For example, the nucleotide containing A always pairs with the nucleotide containing T, and the nucleotide containing C always pairs with the nucleotide containing G. The composition of the second strand therefore depends on the composition of the first strand. Accordingly, the strands are called complementary. This also means that if one knows the nucleotide sequence of one strand, one can automatically infer the sequence of the second strand.

***Transcription***

To convert the information encoded in the DNA of one gene into a protein, the first step is to copy, or transcribe, one of the DNA strands into another nucleic acid molecule called messenger ribonucleic acid (mRNA). This process is performed by specific enzymes in the cell nucleus.

There are different kinds of RNA in the cell that have different functions but the same chemical structure. RNA molecules are similar in their chemical composition to DNA molecules. The main differences are that the sugar component differs between DNA and RNA and that the organic base thymine, which is present in DNA, is replaced by the base uracil (represented by letter U) in RNA. In addition, RNA molecules are single stranded; unlike DNA, they do not have a complementary strand.

During transcription, the DNA sequence representing one gene is converted into mRNA. Only one strand of the double-stranded DNA molecule, however, serves as a template for mRNA synthesis. RNA nucleotides are guided to the DNA sequence that is being transcribed and temporarily bind to it. Again, only one specific RNA nucleotide can bind to each DNA nucleotide (e.g., the RNA nucleotide containing A pairs with the DNA nucleotide containing T, and the RNA nucleotide containing C pairs with the DNA nucleotide containing G). This specificity guarantees that the genetic information contained in the DNA is accurately converted into mRNA. As with the DNA template, the sequence of a triplet of nucleotides in the RNA codes for one amino acid in the final protein.

After all the information for one gene has been copied into an mRNA molecule, the DNA and mRNA molecules separate. The mRNA then undergoes some additional modifications in the cell’s nucleus before it is transported to the cytoplasm for the next step, the translation into the protein product.

***Translation***

In the cell’s cytoplasm, macromolecules called ribosomes attach to, and slide along, the mRNA. In this manner, the ribosomes “read” the sequence of the mRNA’s nucleotide triplets. According to that sequence, the ribosomes recruit a second kind of RNA, the so-called transfer RNA (tRNA) molecules, which guide the amino acids needed for protein synthesis to the mRNA-ribosome complex. One end of each tRNA molecule has a region that recognizes one specific nucleotide triplet on the mRNA. Another region of each tRNA molecule is attached to a specific amino acid. Thus, by recruiting tRNA molecules that recognize the nucleotide sequence of the mRNA, the ribosomes also retain the right amino acids in the right order to form the protein encoded by the gene represented in the mRNA. Specific enzymes then connect the amino acids until the complete protein is synthesized. Because each mRNA molecule can be read consecutively by several ribosomes, many protein molecules can be derived from just one mRNA template.

—Susanne Hiller-Sturmhöfel

The foreign gene product usually can be detected in 10 to 35 percent of the pups. These numbers demonstrate that the creation of transgenic mice is a somewhat inefficient process (Mann and McMahon 1993). Of 100 embryos injected with a foreign gene, only a few will become viable pups that can grow into adult transgenic animals.

Although biochemical tests can determine if the mice that develop from injected embryos (known as first generation, or F_1_, animals) express the foreign gene, the tests cannot determine if all cells of the animal, particularly the germ cells, have integrated the foreign gene. To ensure the gene’s presence in the germ cells, the F_1_ animals are mated with each other. Only F_1_ animals with the foreign gene in their germ cells can pass it on to their offspring (i.e., second generation, or F_2_, animals). The F_2_ animals with the foreign gene will carry it in all their cells. These animals are used to study the gene’s function.

## Applications of Transgenic Mice

As mentioned previously, transgenic animals have not yet been used specifically for alcohol research. The study of transgenic mice created for unrelated research projects, however, has produced unexpected results relevant to alcohol research that offer a glimpse of the potential of this technology.

### Transforming Growth Factor Alpha

Hilakivi-Clarke and colleagues (1992, 1993) studied transgenic mice that express the gene for human transforming growth factor alpha (TGF-alpha) and synthesize excess amounts of TGF-alpha protein. TGF-alpha plays a role during fetal development; excess TGF-alpha, however, can cause liver tumors. The researchers noticed that male transgenic mice expressing human TGF-alpha not only developed liver tumors but also were more aggressive than male nontransgenic animals and displayed altered function of the brain chemical serotonin.

As part of an experiment to study the correlation between aggression and the development of liver tumors in TGF-alpha mice, the animals also received alcohol. When the researchers analyzed different effects of alcohol on the behavior of the transgenic mice, they found that the transgenic mice differed from the nontransgenic mice in their sensitivity to some of alcohol’s effects (Hilakivi-Clarke et al. 1993). For example, the transgenic mice were more sensitive to alcohol’s effects on aggression.

### Corticotropin Releasing Factor

Another existing transgenic mouse line that may have applications in alcohol research carries the rat gene for corticotropin releasing factor (CRF) (Stenzel-Poore et al. 1994). CRF is a hormone involved in the organism’s response to stress. One of the body’s physiological reactions to stress is the synthesis of hormones called glucocorticoids. CRF synthesis is the first step in the chain of events leading to glucocorticoid production. Consequently, CRF is studied in many animal experiments as a physiological measure for the behavioral or emotional condition of stress.

Alcohol alters the body’s reaction to stress. For example, alcohol has been shown to increase the glucocorticoid levels released in response to stress (e.g., Zgombick and Erwin 1988). The CRF mice therefore might provide insight into the mechanisms by which alcohol affects glucocorticoid production levels under stressful conditions.

Both examples illustrate how transgenic animals created for studies unrelated to alcohol research can be used to study phenomena such as physiological sensitivity to alcohol or alcohol’s effects on the response to stress. Other existing transgenic mouse strains might prove to be equally valuable tools in investigating alcohol’s effects and mechanisms of action on different organs.

## Knockout Mice

Whereas transgenic mice carry an additional foreign gene in their DNA, knockout mice are characterized by the targeted elimination of one of their own genes and, consequently, gene products. This approach allows inferences about the function of the deleted gene by comparing the phenotype (i.e., the appearance or behavior) of the knockout mice with that of normal mice. In extreme cases, when the deleted gene has a vital function during embryonic development, knockout mice lacking the gene will not develop beyond a certain embryonic stage (Li et al. 1994; Klein et al. 1993). In the more desirable scenarios, only one specific aspect of metabolism or behavior will be eliminated or modified.

### Creating a Knockout Mouse

As with the starting material for transgenic mice, the gene to be altered in knockout mice also must be identified, isolated, and copied first. In most knockout mice, the targeted gene is not actually deleted from the genome; instead, changes, or mutations, are introduced into the isolated gene, preventing it from encoding a functional gene product. The mutated gene then is transferred into mouse cells.

**Figure 2:**
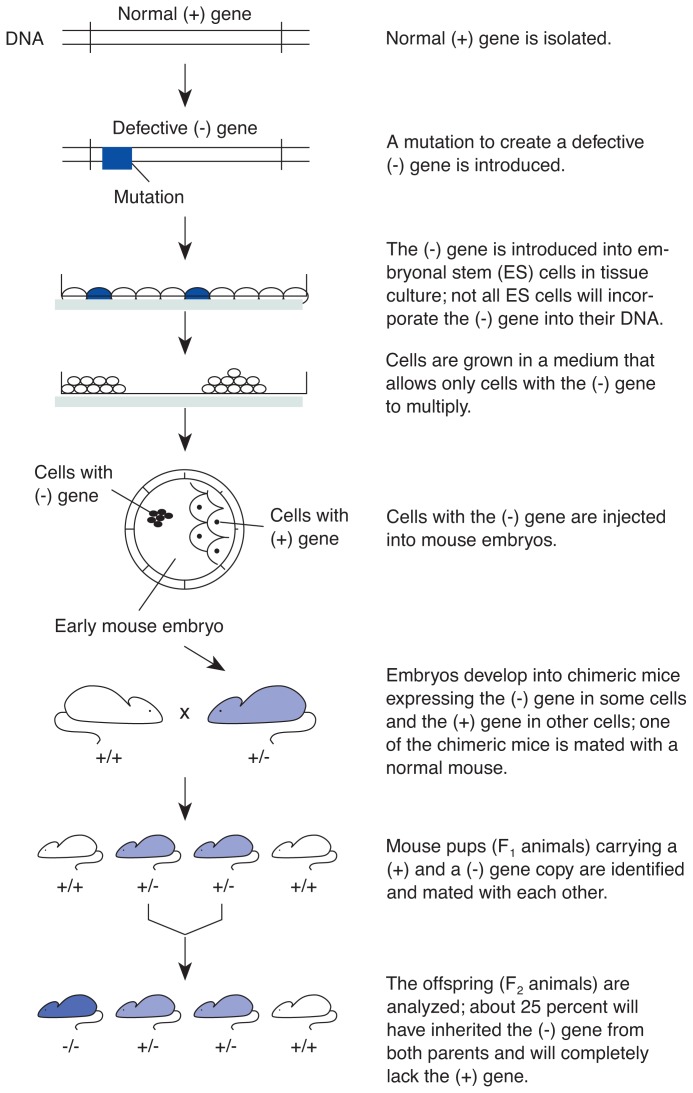
General procedure for the generation of knockout mice.

In contrast with the procedure for transgenic mice, the mutated gene is not introduced directly into an embryo but into a cell type, called embryonic stem (ES) cells (figure 2) (also see Capecchi 1989). These cells can differentiate into all the different cell types and tissues that compose the animal. Once the mutated gene has entered the ES cells, it can exchange places with the cells’ normal gene through a process known as homologous recombination. ES cells expressing the mutated gene are identified by growing the cells in a petri dish in a specific medium in which only the modified cells can survive.

These modified cells then are injected into a mouse embryo at an early stage of development, and the embryo is implanted into a surrogate mother. The injected ES cells and the embryo’s own cells develop together into an intact mouse in which some cells are derived from the ES cells and consequently contain the mutated gene, whereas other cells are derived from the embryo’s own cells and contain the normal gene. Such a mouse is called a chimera and is only the first step in the creation of the real knockout mouse. In the chimera, the mutated gene is not yet present in all the animal’s cells, nor is it integrated in both the maternal and the paternal copies of the gene.

To determine if the chimeras have incorporated the mutated gene into their germ cells, they are mated with normal mice. Pups (i.e., F_1_ animals) in which the mutated gene can be detected must have inherited this gene from the chimeric parent. In addition, they still have an intact gene copy that they have inherited from the normal parent. To replace this copy with a mutated gene, two F_1_ pups are mated with each other. According to the basic laws of inheritance, 25 percent of the F_2_ offspring from this mating will carry two copies of the mutated gene. These F_2_ animals are the knockout mice that are the goal of this long and arduous procedure and whose function or behavior will be analyzed.

### Applications of Knockout Mice

Thus far, the technology of knockout mice rarely has been applied to alcohol research, partly because only a few genes have been identified that may contribute to the susceptibility to alcoholism or that may mediate alcohol’s effects. As researchers’ knowledge increases, however, new applications for knockout mice in alcohol research may become apparent. Already some promising candidate genes have been identified, including neurotransmitters and other molecules that transmit chemical or electrical signals within cells. These substances may play a role in alcohol’s effects on the brain.

#### Gamma-Protein Kinase C

Protein kinases, including protein kinase C (PKC), are enzymes that activate or deactivate the function of proteins by attaching phosphate groups to the proteins. The activation and deactivation of specific proteins is one component of a signaling mechanism through which chemical signals are relayed from the cell’s surface to its interior (Kikkawa et al. 1989). Gamma-PKC is one of several PKC molecules that exist in the body (Kikkawa et al. 1989) and thus also is involved in intracellular signal transmission.

Several lines of evidence suggest that PKC function correlates with alcohol’s effects on the brain. For example, PKC may modify and thus affect the function of a receptor that is located on nerve cells and is activated by the neurotransmitter gamma-aminobutyric acid (GABA). Alcohol researchers have studied the GABA receptor intensely, because it may be responsible for some of alcohol’s effects in the brain. In addition, researchers have found that brain cells containing gamma-PKC are sensitive to alcohol (Palmer et al. 1992).

To study whether gamma-PKC-dependent signal transmission is affected by alcohol, Harris and colleagues (1995) used knockout mice lacking functional gamma-PKC. Because gamma-PKC is not essential during development, the knockout mice developed and reproduced normally. The PKC knockout mice were less sensitive to alcohol’s sleep-inducing effects than were their normal littermates. Also, alcohol’s enhancement of GABA-induced reactions was reduced in brain extracts from knockout mice compared with brain extracts from normal mice. These results indicate that gamma-PKC may be involved in determining an organism’s sensitivity to alcohol.

## Limitations of Transgenic and Knockout Mouse Technology

Although the creation of transgenic and knockout mice is a powerful tool that eventually may provide scientists with a better understanding of human drinking behavior and of alcohol’s effects on the brain and other organs, some limitations exist to the usefulness and validity of such experiments. These limitations are not specific to alcohol research but apply to all research areas analyzing bodily functions and disorders that depend on the cooperation of several genes or that manifest themselves through behavior rather than biochemical reactions.

First, the individual gene being studied must have a large enough impact on the development of the disease (e.g., alcoholism) so that the effect of the gene’s overexpression or deletion can be detected reliably among all other factors contributing to the disorder. Furthermore, other genes sometimes compensate naturally for the function of the deleted gene in knockout mice, thereby masking the deletion’s effect. Accordingly, not all genes and their products can be analyzed using these technologies; the functions of some genes may be identified more readily than the functions of others.

Second, the gene product studied must not be vital to embryonic development; otherwise, too much or too little of it will interfere with normal development. In that case, the transgenic or knockout mouse embryos will not develop to term, and the desired effects (e.g., those resulting from alcohol) cannot be studied.

Third, in transgenic mice, the integration of foreign DNA into mouse DNA to date cannot be targeted to a specific region of the mouse DNA. Consequently, the foreign DNA may integrate in the middle of another gene and thus disturb that gene’s function (Lacy et al. 1983). Researchers should be aware of this possibility when interpreting their findings.

Fourth, similar to the site of foreign DNA integration, the amount of the foreign gene and its product in each cell cannot be predicted. The effect of the foreign gene on the transgenic animal may vary, depending on how much of the gene product is made. Similarly, in many cases the foreign gene will be expressed in all tissues, in contrast with the normal physiological condition, in which the gene may be expressed only in selected cells (Lacy et al. 1983). To circumvent this problem, scientists currently are developing methods that target the expression of foreign genes to the cells in which they normally are active. This process uses specific regulators that allow gene expression only in specific cell types or tissues.

Fifth, the choice of mouse lines used to create transgenic or knockout mice may affect the experiments’ results. Just as two people may respond differently to alcohol although they share some of the same genes that mediate alcohol’s effects, two mouse lines may react differently to the addition or deletion of the gene to their genetic material (Grant et al. 1992; Liu et al. 1993).

Nonetheless, these caveats do not diminish the potential that transgenic and knockout mice hold for alcohol research; they illustrate, however, that the results obtained with genetically engineered animals should be interpreted cautiously, because they may not be as straightforward as they initially seem.

## Antisense RNA Strategies

Another method for exploring the role of specific genes in mediating alcohol’s effects in a living organism is antisense RNA technology (Crooke 1992). Similar to the approach using knockout mice, antisense RNA technology reduces or prevents the expression of a specific gene. In contrast with the knockout mice, however, this modification usually is not complete or permanent, because the antisense RNA can be administered to the animals temporarily.

Antisense RNA technology has several potential advantages compared with transgenic and knockout mice. For example, antisense RNA treatment is faster and cheaper than creating and breeding genetically modified mice. Because it is not a permanent modification, antisense RNA treatment also avoids some of the limitations of knockout and transgenic mice, such as the difficulty of modifying genes that are critical during development. With antisense RNA technology, the animals can develop normally before their gene expression is manipulated. Finally, antisense RNA may have some therapeutic potential in humans, for example, by targeting the receptors for certain neurotransmitters in the brain, provided that adequate amounts of RNA can be delivered to brain cells (Crooke 1992).

### What Is Antisense RNA Treatment?

**Figure 3:**
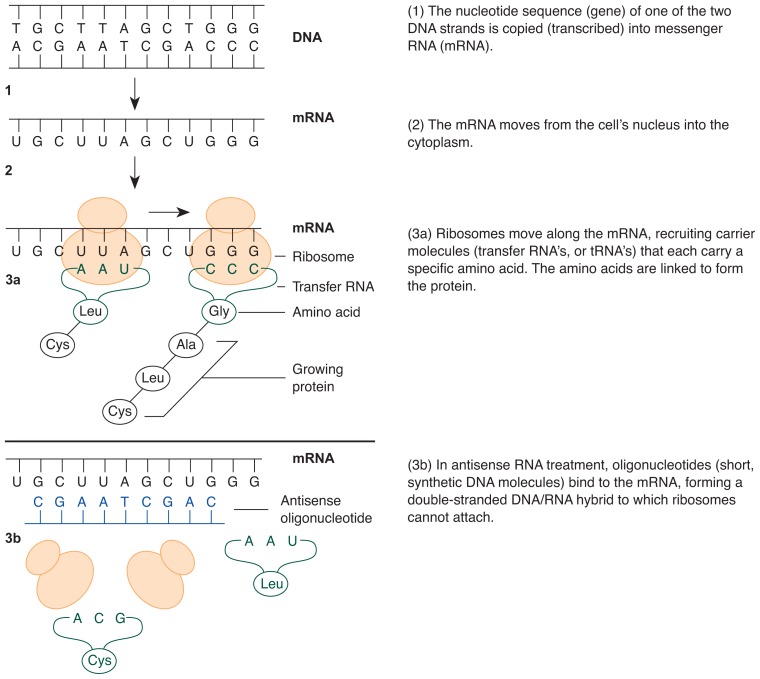
The conversion of genetic information into protein without and with antisense RNA treatment. Steps 1–3a show the usual way in which the information on a DNA strand serves as a blueprint for generating proteins. In antisense RNA treatment (3b), a “dummy” sequence of DNA prevents ribosomes from carrying out the process of making proteins. Using this technique, researchers may be able to investigate the link between genes and alcohol-related problems. For example, certain proteins may be needed to manufacture neurotransmitters involved in the desire to consume alcohol; if blocking the creation of one of those proteins would change alcohol consumption, the gene(s) responsible for making that protein might be involved in the urge to drink alcohol.

The conversion of the genetic information encoded in the DNA into a protein product is a complex process. Briefly, through a process called transcription, the DNA information in the cell’s nucleus is copied into an intermediary molecule, the messenger RNA (mRNA). The mRNA is transported to the cell’s cytoplasm, where it serves as a template for the synthesis of a protein in a process referred to as translation (for more information on the steps involved in transcription and translation, see figure 3 and the sidebar on p. 208).

Antisense RNA technology aims to inhibit the translation of an mRNA into its respective protein. As with transgenic and knockout mice, the technique requires that the gene to be studied has been isolated and copied and that its exact DNA sequence is known. During antisense RNA treatment, however, the gene itself is not the target of the procedure. Instead, the target is the mRNA transcribed from the gene.

To block translation of the mRNA, scientists make a short synthetic DNA molecule called an oligonucleotide, which can bind to the end of the mRNA molecule at which translation begins. This oligonucleotide is called the “antisense” molecule because it is complementary (i.e., like a reverse image) to a section of the “sense” information encoded in the mRNA. When the DNA oligonucleotide is introduced into a cell, it can attach itself to the complementary region of the mRNA, creating a region that is a DNA–RNA hybrid (figure 3). This DNA–RNA hybrid region interferes with translation of the mRNA, because the proteins required for this process bind only to the free RNA molecule, not to a DNA–RNA hybrid. In addition, the DNA–RNA molecules degrade rapidly in the cell. Consequently, no protein is synthesized from the mRNA.

This mechanism works in isolated cells, tissues, and whole animals. The oligonucleotide, which is readily taken up by individual cells, can be delivered to cells and tissues by various methods (Wahlestedt 1994). It can be administered to animals by a one-time injection or over a longer period by chronic transfusion. Cells in specifically targeted organs or regions of the body incorporate the oligonucleotide. By varying the length or frequency of oligonucleotide administration, researchers can modify the duration for which the targeted protein is not being synthesized. They also can study the effects of this manipulation at different developmental stages and under different experimental conditions (e.g., in the presence or absence of alcohol). Consequently, antisense RNA technology allows a flexibility in studying the effects of individual genes that cannot be achieved with transgenic or knockout mice in which the genetic material is modified permanently.

### Applications of Antisense RNA Technology

Antisense RNA strategies have not yet been applied specifically to alcohol research. However, the functions of several neurotransmitters and their receptors, both of which may contribute to alcohol’s effects on the brain, have been studied under experimental conditions not related to alcohol.

#### *N*-methyl-D-aspartate Receptor

One important neurotransmitter in the brain is the amino acid glutamate, which binds to a receptor on the nerve cell surface called the *N*-methyl-D-aspartate (NMDA) receptor. Alcohol researchers study this receptor because its response to glutamate binding is thought to contribute to alcohol withdrawal seizures.

The NMDA receptor also has other effects on the brain that are not related to alcohol. For example, its response to glutamate binding may be involved in causing cell death after ischemic strokes in the brain. An ischemic stroke occurs when obstruction of a blood vessel disrupts the blood flow to an area of the brain. Such a stroke can lead to the death of the brain cells in that area.

When isolated brain cells were treated with antisense oligonucleotides that could bind to the NMDA receptor mRNA, the number of NMDA receptors on the cells decreased, and cell death could be induced only to a lesser extent than in untreated cells (Wahlestedt et al. 1993). Similarly, in animals treated with the antisense oligonucleotides, the number of cells that died after the obstruction of a brain artery also was reduced significantly (Wahlestedt et al. 1993). These findings demonstrate that the NMDA receptor does in fact contribute to cell death after ischemic strokes. In addition, the example shows that antisense RNA technology can affect the expression of genes both in isolated cells and in whole animals, thereby allowing researchers to study the function of these genes in both experimental systems.

## Limitations of Antisense RNA Technology

One major advantage of antisense RNA technology—that it is a temporary, easy-to-manipulate pharmacological approach—also contributes to the technology’s limitations. Some of these limitations are as follows:

- Delivering sufficient amounts of antisense oligonucleotides to tissues in living animals, especially to the brain, can be difficult. As a result, the inhibition of a gene’s function in an animal or even in a specific tissue rarely is complete.
- Antisense oligonucleotides only have a limited lifespan in the body before they are broken down (Whitesell et al. 1993). Researchers are trying to modify the chemical structure of the oligonucleotides to extend that lifespan, but a specific treatment protocol probably will need to be determined empirically for each gene or gene product to be studied.
- The lifespans of the mRNA and the protein being studied must be established for each experiment, because they determine the duration of the antisense treatment required to produce meaningful results.
- Researchers must ensure that the oligonucleotides bind only to the desired mRNA and do not interfere with the translation of other mRNA’s in the cell. Such an unwanted interaction could distort the results and lead to misinterpretations of the gene’s function.
- Oligonucleotide treatment in some instances can be toxic to cells or to living animals (Wahlestedt 1994).

## Summary

This article presents three exciting new technologies that allow scientists to study the functions of single genes in the context of the living organism. Because these techniques specifically examine the functions of individual genes, they appear particularly well suited for the analysis of genes involved in the development of polygenic disorders such as alcoholism.

So far, these technologies have not been applied systematically to studying the causes and effects of alcoholism. This partly is attributable to the fact that researchers have isolated few candidate genes that may contribute to the development of alcoholism or to mediating alcohol’s effects. As some of the examples in this article demonstrate, however, even seemingly unrelated experiments may produce results relevant to alcohol research. At the very least, the examples illustrate the potential of new genetic engineering technologies in animal models to help scientists answer pressing questions in many research areas.

## Footnotes

1 For a definition of this and other technical terms used in this article, see central glossary, pp. 182–183.
